# *Salmonella* Typhimurium Triggers Extracellular Traps Release in Murine Macrophages

**DOI:** 10.3389/fcimb.2021.639768

**Published:** 2021-04-26

**Authors:** Amy Mónaco, Nicole Canales-Huerta, Jorge Jara-Wilde, Steffen Härtel, Jose Alejandro Chabalgoity, María Moreno, Paola Scavone

**Affiliations:** ^1^ Departamento de Desarrollo Biotecnológico, Instituto de Higiene, Facultad de Medicina, Universidad de la República, Montevideo, Uruguay; ^2^ Laboratorio de Análisis de Imágenes Científicas SCIAN-Lab, Integrative Biology Program, Institute of Biomedical Sciences ICBM, Faculty of Medicine, University of Chile, Santiago de Chile, Chile; ^3^ Biomedical Neuroscience Institute BNI, Faculty of Medicine, University of Chile, Santiago de Chile, Chile; ^4^ Centro de Informática Médica y Telemedicina CIMT, Faculty of Medicine, University of Chile, Santiago de Chile, Chile; ^5^ National Center for Health Information Systems CENS, Santiago de Chile, Chile; ^6^ Departamento de Microbiología, Instituto de Investigaciones Biológicas Clemente Estable, Montevideo, Uruguay

**Keywords:** *Salmonella*, macrophages, extracellular traps, MET, defense mechanism

## Abstract

*Salmonella* comprises two species and more than 2500 serovars with marked differences in host specificity, and is responsible for a wide spectrum of diseases, ranging from localized gastroenteritis to severe life-threatening invasive disease. The initiation of the host inflammatory response, triggered by many Pathogen-Associated Molecular Patterns (PAMPs) that *Salmonella* possesses, recruits innate immune cells in order to restrain the infection at the local site. Neutrophils are known for killing bacteria through oxidative burst, amid other mechanisms. Amongst those mechanisms for controlling bacteria, the release of Extracellular Traps (ETs) represents a newly described pathway of programmed cell death known as ETosis. Particularly, Neutrophil Extracellular Traps (NETs) were first described in 2004 and since then, a number of reports have demonstrated their role as a novel defense mechanism against different pathogens. This released net-like material is composed of cellular DNA decorated with histones and cellular proteins. These structures have shown ability to trap, neutralize and kill different kinds of microorganisms, ranging from viruses and bacteria to fungi and parasites. *Salmonella* was one of the first microorganisms that were reported to be killed by NETs and several studies have confirmed the observation and deepened into its variants. Nevertheless, much less is known about their counterparts in other immune cells, e.g. Macrophage Extracellular Traps (METs) and *Salmonella*-induced MET release has never been reported so far. In this work, we observed the production of METs induced by *Salmonella enterica* serovar Typhimurium and recorded their effect on bacteria, showing for the first time that macrophages can also release extracellular DNA traps upon encounter with *Salmonella* Typhimurium. Additionally we show that METs effectively immobilize and reduce *Salmonella* survival in a few minutes, suggesting METs as a novel immune-mediated defense mechanism against *Salmonella* infection. Of note, this phenomenon was confirmed in primary macrophages, since MET release was also observed in bone marrow-derived macrophages infected with *Salmonella*. The evidence of this peculiar mechanism provides new incipient insights into macrophages´ role against *Salmonella* infection and can help to design new strategies for the clinical control of this transcendental pathogen.

## Introduction

*Salmonella* comprises two species and more than 2500 serovars with marked differences in host specificity; and is responsible for a wide spectrum of diseases, ranging from localized gastroenteritis to severe life-threatening invasive disease (Majowicz et al., 2010). It is generally transmitted to humans through consumption of contaminated food, or through fecal-oral route (Betancor et al., 2010; Gopinath et al., 2012; Bonardi et al., 2017). Even when salmonellosis cases are mild, it can be lethal, depending mainly on the particular infecting serotype and immunological status of the host. *Salmonella* has evolved multiple mechanisms, comprising a sophisticated repertoire of virulence factors, to compete with the gut microbiota for access to the host cells and successfully infect them, thereby avoiding or subverting host immune responses (Shea et al., 1996). A distinctive feature of *Salmonella* pathogenesis is the capacity to invade and live inside professional phagocytic cells inside *Salmonella* containing vacuoles (SCV). Particularly, macrophages represent an ideal niche for this purpose, whereas other innate immune cells such as neutrophils kill bacteria through oxidative burst, among other mechanisms (Burton et al., 2014). The initiation of an inflammatory response, triggered by many Pathogen-Associated Molecular Patterns (PAMPs) that *Salmonella* possesses, recruits neutrophils and inflammatory monocytes, in order to restrain the infection (Pham and McSorley, 2015). Besides the classical phagocytic role, these recruited cells have an additional mechanism for controlling bacteria that relies on the release of extracellular traps (ETs), a newly described pathway of programmed cell death known as ETosis. This released net-like material is composed of cellular DNA decorated with histones and cellular proteins (Brinkmann et al., 2004). These trapping structures have shown ability to neutralize and kill all kinds of microorganisms, ranging from viruses and bacteria to fungi and parasites (Brinkmann et al., 2004; Urban et al., 2006; Saitoh et al., 2012; de Buhr et al., 2018). Their relevance during pathogen elimination becomes apparent by the clinical finding that hereditary NET deficiency leads to invasive aspergillosis, that is later controlled by restoration of NET formation ability with gene therapy (Bianchi et al., 2009). Regarding *Salmonella*, it was the first microorganism that was reported to be killed by NETs, and since, several other studies have confirmed it and deepened into its variants (Brinkmann et al., 2004; Pieper et al., 2017). ETosis has been also attributed more recently to macrophages [reviewed in (Doster et al., 2018)] *Salmonella*-induced MET release, however, has never been reported so far, although other enterobacteriaceae family members as *E. coli* are known inducers (Webster et al., 2010). In this work, we observed the production of METs induced by *Salmonella enterica* serovar Typhimurium and recorded their effect on bacteria. Our results show for the first time that macrophages release extracellular traps upon *Salmonella* infection, where bacteria are retained and killed. This suggests a new defense mechanism against these highly prevalent bacteria. Although these results are preliminary they provide new insights in macrophages' role against *Salmonella* infection.

## Materials and Methods

### Bacterial Strains and Cell Lines

*Salmonella enterica* serovar Typhimurium LVR01 (*aroC-*) (Chabalgoity et al., 2000), was used in the present study. For confocal microscopy experiments, a GFP constitutive expressing derivative, LVR01-gfp-rpsM, was used instead. This derivative was constructed by phage P22 transduction with a lysate kindly provided by Dr. Isabelle Hautefort (Institute of Food Research, Norwich, United Kingdom) (Hautefort et al., 2003).

The murine J774A.1 macrophage-like cell line was obtained from American Type Culture Collection (ATCC^®^ TIB67™) and maintained in Dulbecco’s Modified Eagle’s Medium (DMEM), supplemented with 10% of heat inactivated fetal bovine serum (hiFBS). Primary bone marrow-derived macrophages (BMDM) were isolated from femurs of C57BL/6 mice killed by cervical dislocation. The bone marrow was flushed out with Roswell Park Memorial Institute (RPMI) 1640 medium supplemented with 10% (v/v) FBS and 2 mM glutamine, and for maintenance of BMDM in culture this medium was further supplemented with 20% (v/v) of supernatant taken from L929 cells (a murine M-CSF-producing cell line) (Royle et al., 2003). All procedures were carried out in accordance with the local guidelines and approved by the Comisión Honoraria de Experimentación Animal, Uruguay.

### Macrophage Culture and Infection

Macrophages were seeded in 24-well plates (Greiner bio-one, Frickenhausen, Germany) to a density of 3x10^5^ cells per well and let adhere for 2hs. *Salmonella* LVR01-gfp-rpsM was diluted in the appropriate culture medium and added to the cells at a MOI 1, 10, 40 or 100:1, depending on the experiment. Incubation was performed at 37°C in 5% CO_2_ atmosphere for 10, 30 or 60 minutes. DNase I (100 U/ml) (Sigma-Aldrich, MO, USA) was added 30 minutes prior to infection. As a positive control for MET release, phorbol myristate acetate (PMA) 200 μM (Sigma-Aldrich, MO, USA) was used (Aulik et al., 2012). To evaluate whether DNAse I was toxic for bacteria, control wells with bacteria but no cells were exposed to the same assay conditions. For sterile MET production assessment, cells were cultured after 3 hours (2 hours adhesion + 1 hour condition), supernatants were removed and transferred to a fresh culture (2 hours adhesion). As a control, in a different well the cell supernatant was removed after 2 hours of adhesion and substituted with fresh medium. For time lapse video recording, 27 mm glass base dishes (Thermo Fisher Scientific, Waltham, Massachusetts, USA) were used instead of the 24-well plates described above. For bacterial cytotoxicity assays DNAse I was added 30 minutes prior to infection. Cells were lysed with Triton X-100 0.1% (Sigma-Aldrich, MO, USA) and bacterial dilutions were performed for appropriate count on LBA plates. Percentage of bacterial death was determined as: [(inoculum – ufc recovered)/inoculum]x100.

### Staining, Confocal Microscopy Image Acquisition and Processing

Observation was carried out using a Zeiss LSM 800-AiryScan confocal laser scanning microscope (Carl Zeiss AG; Jena, Germany), with an inverted Axio-observer 2.0 stative, and Zen Blue 2.3 Software. In order to determine different aspects of MET production, slides were fixed overnight with 4% paraformaldehyde and then washed with phosphate-buffered saline (PBS). Next, a permeabilization step was performed with 0.3% Triton X-100 in Non-Permeabilization (NP) buffer for 20 minutes at room temperature (RT). The staining was performed as follows: 1:100 dilution of 1 mg/ml Hoechst 33342 (Molecular Probes, Thermo Fisher Scientific, Waltham, Massachusetts, USA) for DNA staining and Wheat Germ Agglutinin (WGA) - Texas Red (Molecular Probes, Thermo Fisher Scientific, Waltham, Massachusetts, USA) for membrane staining in NP buffer (BSA 2%, NH_4_Cl 50mM in PBS), during 30 minutes at room temperature. Once staining was finished, slides were washed and mounted with ProLong™ Gold Antifade (Thermo Fisher Scientific, Waltham, Massachusetts, USA). 3D image stacks were acquired using 350/460, 488/520 and 595/615 nm excitation/emission wavelengths, a 63X oil immersion objective (NA = 1.4), 0.5 µm step size in the z axis, and 1024 × 1024 pixels in the x-y plane with 99 nm pixel size. The 3D image stacks were analyzed using ImageJ/FIJI software (Schindelin et al., 2012). Color, contrast and γ-adjustments were performed for each fluorescence channel and z-slice individually prior to z-projection. For *in vivo* recording, the same staining was performed and the acquisition parameters were adapted to have the fastest possible acquisition. Movies were recorded during 3 minutes, at 0.57 seconds per frame for **Movie 1**, and 0.49 seconds per frame for **Movie SM2**. Images were deconvolved with Huygens Scripting Software (SVI; Hilversum, The Netherlands), using the Classical Maximum Likelihood algorithm with custom parameters. Signal to noise ratio (SNR 5) was manually adjusted until the deconvolved images were free of pixel noise. The segmentation routines applied were developed at one of the authors’ laboratory (SCIAN-Lab, www.scian.cl) on the basis of IDL 7.1.2 (Harris Geospatial; Broomfield, CO, USA). For the segmentation of the bacteria we first applied the Median filter with window width 4-5 pixels. This is a smoothing filter that replaces each pixel value with the median of a two-dimensional neighborhood (square pixel window) of a given width. The filtered image was manually binarized by setting an intensity threshold value, used to convert each image pixel by comparing its intensity against the threshold value, setting the pixel to 0 (lower than the threshold value) or 1 (equal or greater than the threshold value). Nucleus segmentation was performed with the Median filter and binarized manually by setting an intensity threshold producing an initial set of regions of interest (ROIs, one per nucleus). Afterwards, morphological filtering (“Fill/remove”) was applied to fill ROI holes and filter out small spurious (non-nuclear) ROIs. Afterwards, an Active contour algorithm with Gradient vector flow (Xu and Prince, 1998; Fanani et al., 2010) was applied to optimize the final shape of each nucleus. The segmentation of the cytoplasm was done by applying an intensity threshold (manually set for each slice) and the Fill/remove filter. The deconvolved images of individual bacterium were tracked semi-automatically using TrackMate (Tinevez et al., 2017), an open source software plugin for FIJI for automated, semi-automated, and manual tracking of single-particles. This plugin displays the tracks based only on their links, and lays out spots from bottom to top according to time. Each branching event generates a new vertical lane. The segmentation, filtering, and particle-linking processes and results were visualized immediately in 2D or 3D, to judge their efficiencies and to adjust their control parameters. Bacterial tracking was performed in the GFP channel (bacteria signal). The ROIs were segmented and the tracking recorded.

### DNA Quantification

J774A.1 macrophages were incubated for 30 minutes with PMA or LVR01, with or without DNAse I pretreatment as above indicated; all dilutions were performed in 1X TE buffer. A 1:200 dilution of Quant-iT PicoGreen dsDNA reagent (cat. P7581, Invitrogen, CA, USA) was added to an equal volume of the samples. Fluorescence was determined within 10 minutes with 485/520 nm excitation/emission wavelengths, using an automated plate reader (FLUOstar OPTIMA). A standard curve with total DNA extracted from J774A.1 cells was included. DNA quality and quantity was assessed by spectrophotometric measurements at 260/280 nm using NanoDrop 2000 (Thermo Fisher Scientific; Waltham, MA, USA) after extraction with DNeasy Blood & Tissue kit (QIAGEN; Hilden, Germany).

### Statistical Analysis

The statistical significance of differences between conditions was analyzed using Student-T test with Prism 5 software (GraphPad Software; San Diego, CA). In each case a value of p<0.05 was considered as statistically significant.

## Results

**Figure 1:**
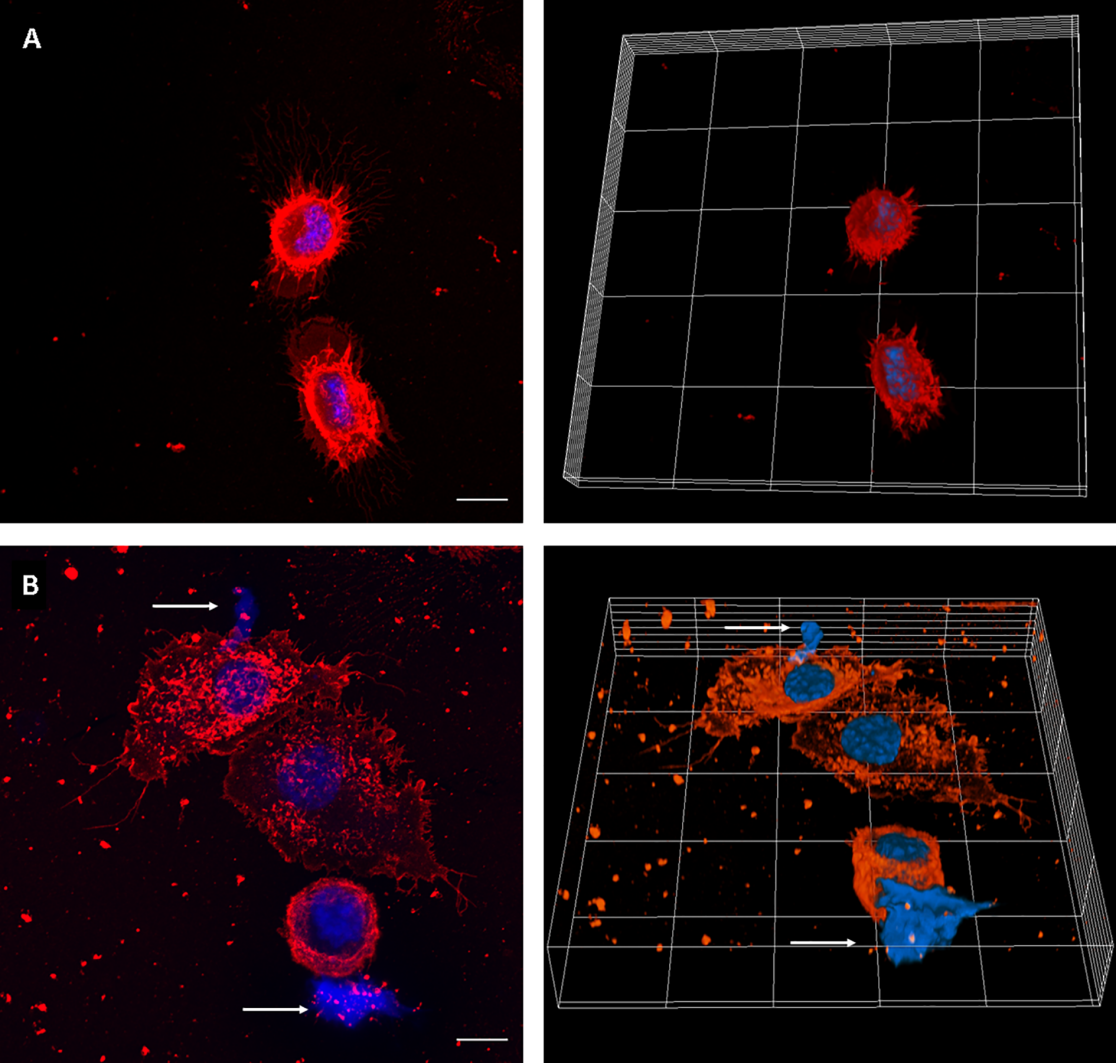
MET release in PMA-treated J774A.1 macrophages. Confocal microscopy images of **(A)** Unstimulated cells, and **(B)** cells incubated with PMA (200 µM) for 30 minutes. The left panels show maximum intensity z-projection of the image stack; the right panels show 3D models of cells. WGA staining is shown in red and DNA in blue. The arrows point out the presence of METs. The scale bar represents 10 µm.

We first set up the conditions to evaluate MET release by J774A.1 macrophages, using uninfected and PMA-treated cells as negative and positive controls, respectively. MET release was not observed in untreated macrophages. Instead, PMA addition induced release of MET, as identified by fluorescent staining with the DNA-binding dye Hoechst, and laser scanning confocal microscopy (**Figure 1**).

**Figure 2:**
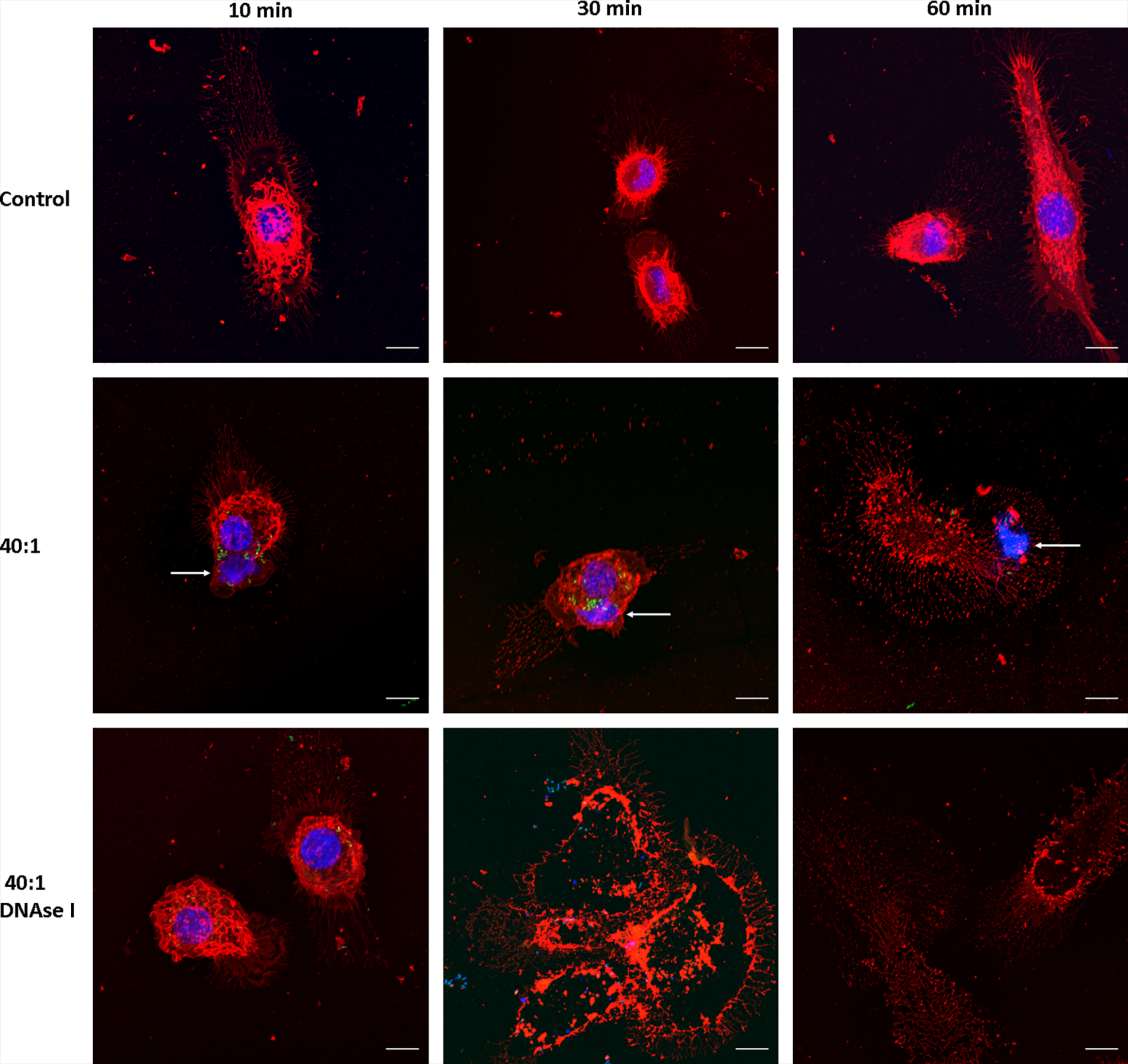
MET release after *Salmonella* infection. Confocal microscopy images of J774A.1 macrophage extracellular trap release at t = 10, 30, and 60 min. Upper row: Uninfected cells (Control). Central row: Cells infected with *Salmonella enterica* serovar Typhimurium LVR01 (40:1). Bottom row: Cells infected with LVR01 pretreated with DNAse I. Maximum intensity z-projections are shown for membrane visualization with WGA staining (red), DNA (blue), and *Salmonella* (green). The arrows point out MET release. The scale bar represents 10 µm.

We then evaluated whether *Salmonella* induces MET formation in J774A.1 macrophages. Infection was carried out at a high MOI as a proof of concept, 40:1 (bacteria:cells ratio), and followed up for 1 hour (t = 10, 30 and 60 minutes) after infection. We found MET release after *Salmonella* infection; average size of the fibers 16.7 ± 10 μm, ranging between 6.6 and 50.3 μm (**Figure 2**). MET release was observed as fast as 10 minutes after infection (**Figure 2**, central row, left panel), and by 30 minutes we still observed METs and macrophages in normal shapes (**Figure 2**, central row, central panel). After 60 minutes of infection, most macrophages were no longer viable as inferred from the lack of defined membrane and nucleus, as well as the presence of cellular debris (**Figure 2**, central row, right panel). MET structures were observed beyond cellular boundaries, close to the macrophages (**Figure 2**, central row, left panel). They presented uniform organization compared to a normal nucleus, revealed by the intensity of the staining. As the Hoechst 33342 dye binds to the minor groove of the DNA at AT-rich sequences differentiates distinct degrees of chromatin condensation state. The nuclear DNA with different zones of compaction is reflected by different degrees of Hoechst intensity as more intense is the fluorescence more condensed is the chromatin, while the METs that mainly consist of DNA fibers and enzymes are observed as an even blue color.

Since METs are mainly composed of DNA, we confirmed the nature of this process by inducing them in the presence of DNAse I (**Figure 2**, bottom row). METs were not observed when macrophages were pre-treated with DNAse I, confirming that DNA is the major structural component of these fibers. Moreover, cell damage was still observed, even in the presence of DNAse I, suggesting that the process of ETosis was taking place but METs were no longer visualized because DNAse I had already digested them. Another clue that this phenomenon is related to ETosis is the presence of several enucleated cells observed 30 minutes after infection (**Figure 2**, bottom row, central panel).

**Figure 3:**
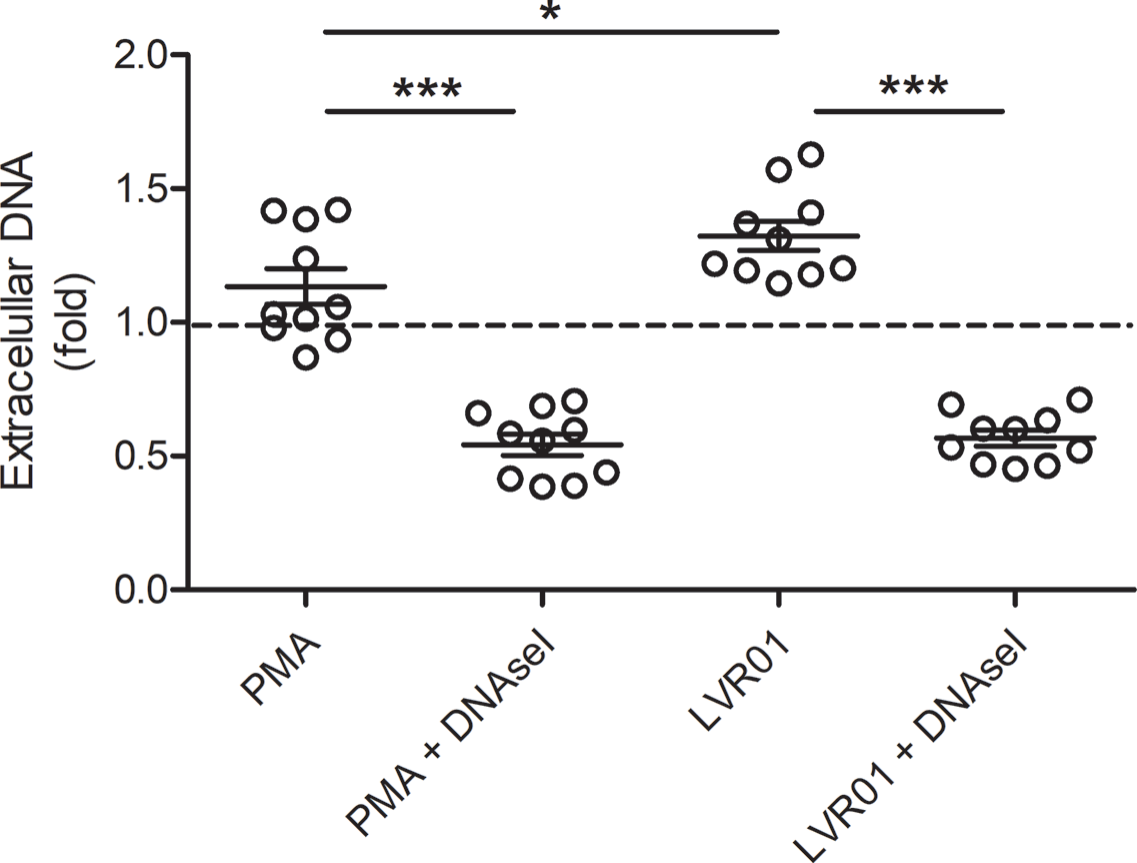
Extracellular DNA quantification. Cells were incubated for 30 minutes with PMA or LVR01, with or without DNAse I pretreatment. DNA quantification was assessed with PicoGreen reagent. The dashed line indicates mean value of untreated cells. Results are shown as fold increase of individual replicates (n = 10) and mean ± SD for each evaluated condition. *p ≤ 0.05 and ***p ≤ 0.001, Student T-test. One representative experiment of 6 independent ones performed.

Extracellular DNA was quantified 30 minutes after MET release induction (**Figure 3**), showing that. *Salmonella* infection significantly increased extracellular DNA. DNAse I pre-treatment resulted in a significant decrease of quantified DNA, confirming that a significant amount of DNA was exposed to DNAse I activity, hence outside cell boundaries.

**Figure 4:**
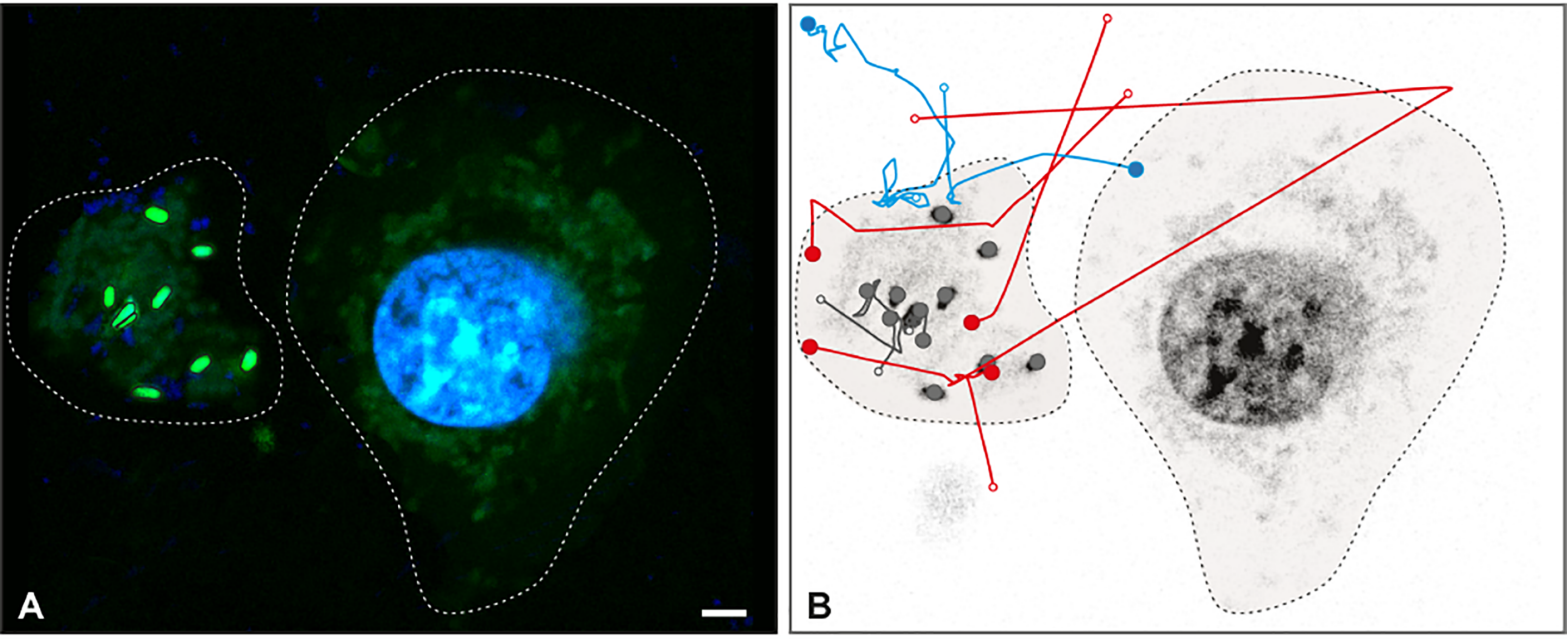
*Salmonella* trapped in MET of J774A.1 macrophages. Live imaging was performed by confocal microscopy and *Salmonella* were tracked by image analysis. **(A)** Fluorescent image at t = 0 s with DNA (blue) and *Salmonella* (green) channels shows a J774A.1 macrophage (dashed line on the right) and a MET (dashed line on the left). Within the MET, 9 bacteria with high intensity values were identified. **(B)** Inverse grey scale color table for DNA channel is shown for t = 231 s together with the 15 tracked trajectories for *Salmonella.* The positions of each bacterium at the beginning and the end of the trajectory are marked by an open and closed circle, respectively. Trajectory lines are colored according to bacteria status: trapped within the MET (grey); free and later trapped (red) and transiently trapped (blue). Trajectories of swimming bacteria are not displayed. The scale bar represents 10 µm.

Live imaging proved that *Salmonella* became trapped in METs (**Movie 1**). Once trapped, bacteria stopped moving after 30 seconds of efforts to escape (**Movie 1**, i.e. minute 01:30.213 to 02:00.463). The trajectories of *Salmonella* tracked from **Movie 1** are shown in **Figure 4**. The analysis revealed that 11 bacteria were trapped and immobilized within the MET from the beginning of the movie (grey trajectories); 4 bacteria were detected swimming in the media and trapped within the MET, where they stayed until the end of the movie (red trajectories); 4 bacteria were trapped temporarily within the MET and then escaped the trap (blue trajectories), and a total of 72 swimming *Salmonella* entered and left the movie frame transiently without getting in contact with the MET (trajectories not shown). Tracking analysis also revealed that some bacteria were constrained to one particular area of the image, which corresponds to a MET (Still image 2, **Movie 2**). These trapped bacteria showed confined displacements of only a few microns (**Movie 2**); and compared to the free bacteria, they have less movement.

Once bacteria were trapped, the GFP started to progressively diminish until the end of the movie acquisition. A GFP intensity decrease was observed by a time-lapse total fluorescence plot (data not shown). Swimming *Salmonella* did not show an intensity decrease after being exposed to the highest laser power for 3 minutes (data not shown). In another approach to visualize this phenomenon, we processed **Movie 1** so as the emission color of the channel was changed to a red/green lookup table (LUT) (**Movie SM1**) where high fluorescence intensity values are observed in green and low intensity values in red. Besides, the same LUT was applied for both green and blue channel (for GFP and DNA, respectively), as bacteria were stained with both fluorophores. This allows observing that most trapped bacteria undergo a switch from green to red, indicating a decrease in fluorescence; whereas DNA signal from the nucleus remains constant (bluing one in **Movie 1**). This last observation rules out that GFP dimming is due to photobleaching.

**Figure 5:**
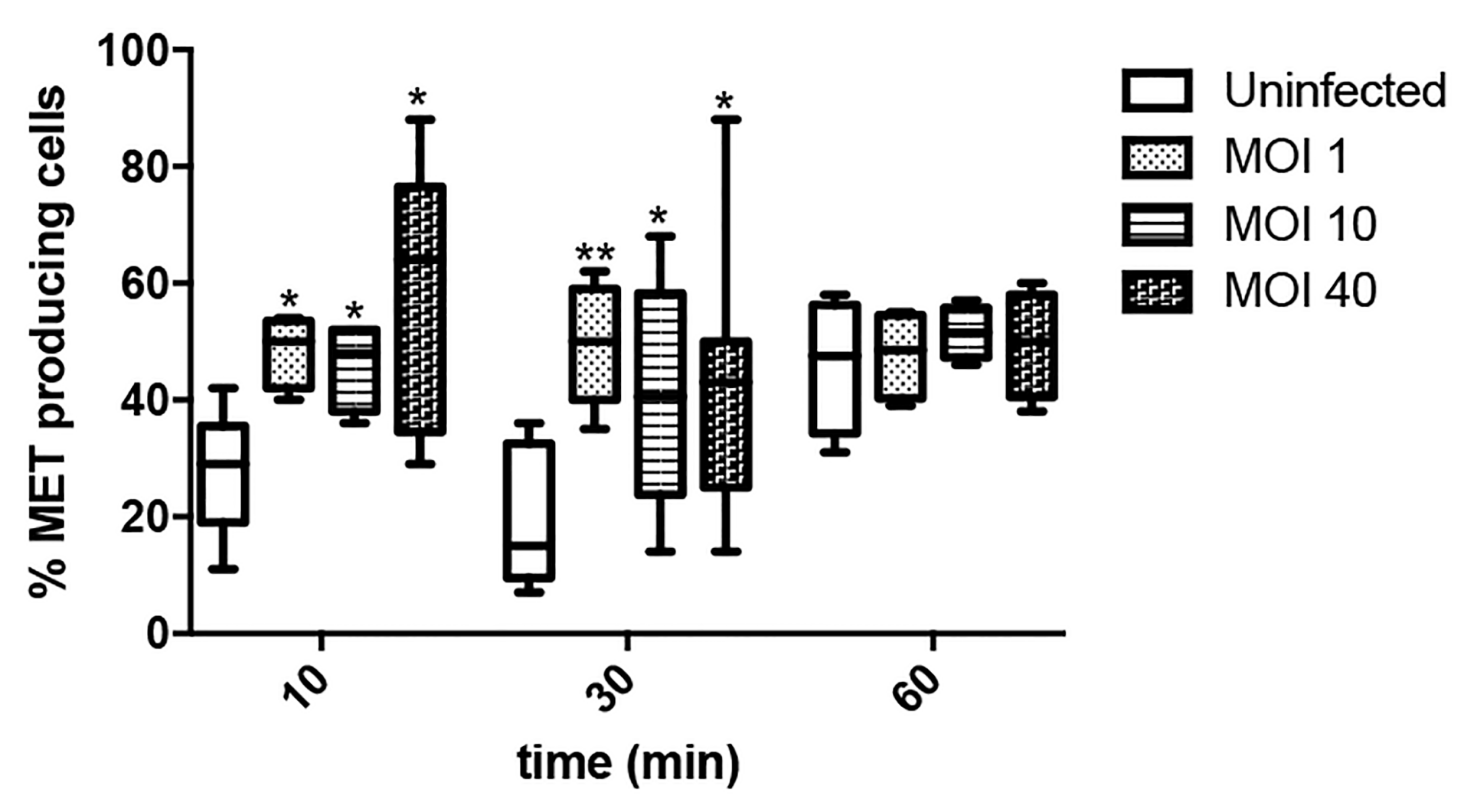
Percentage of ET releasing macrophages after *Salmonella* infection. Cells were infected for 10, 30 or 60 minutes with *Salmonella*, MOI 1, 10 or 40. Number of selected fields was established so as to allow counting a minimum of 150 cells in each condition. Results are shown as mean ± SEM for each evaluated condition. * indicates significant differences between infected and uninfected cells in the same timepoint, *p ≤ 0.05, **p ≤ 0.01, Student T-test. One representative experiment of 2 independent ones performed.

We then assessed through time the percentage of MET releasing cells at different MOIs, ranging from low (1) to high (40), in order to evaluate whether the process was influenced by bacterial load. As shown in **Figure 5**, *Salmonella* infection rapidly increases the number of cells undergoing MET release, irrespective of the multiplicity of infection. Noteworthy, by 10 and 30 minutes the percentage of MET releasing cells is similar, while by 60 minutes uninfected cells exhibit comparable MET induction to *Salmonella* infected cells. This could be explained by the fact that minimum amount of extracellular material passively released by uninfected cells (e.g. in case of cell death) is sufficient to act as a DAMP (damage-associated molecular pattern) that triggers a proinflammatory cascade that includes cytokines as IFNγ or TNFα, both which are known MET inducers (Mohanan et al., 2013; Wong and Jacobs, 2013). In this way, sterile MET production could still be induced, amplifying the effect even when no infection is occuring. Indeed, this hypothesis was confirmed by the observation of MET release by fresh cells after the addition of supernatant from another culture, whereas this event was not observed after substituting supernatant with fresh medium (**Supplementary Figure S1**).

**Figure 6:**
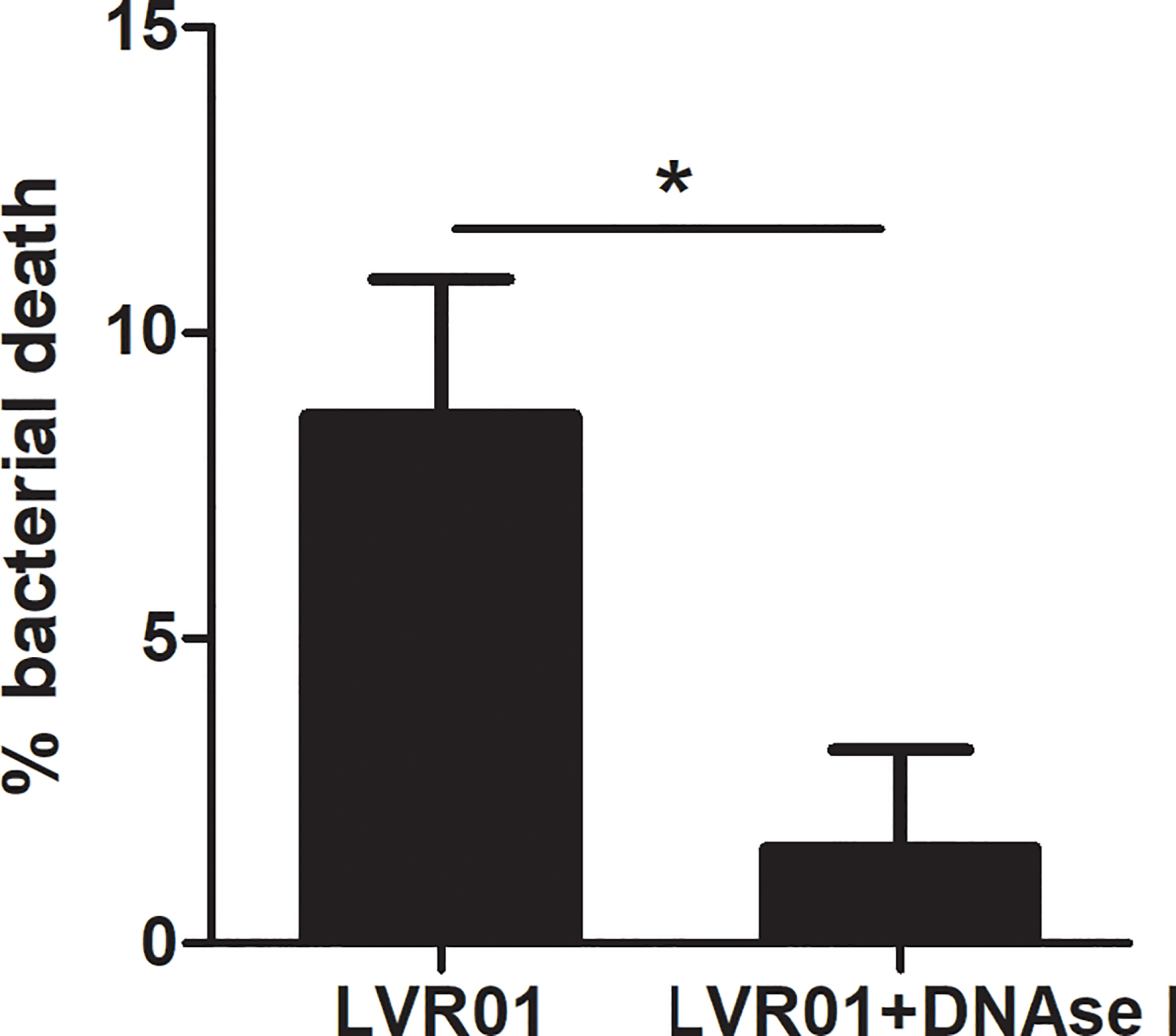
Percentage of *Salmonella* death by METs. *Salmonella* was co-cultured for 60 minutes with J774A.1 cells, in presence or absence of DNAse I. Cells were lysed and bacteria counted. Results are shown as mean ± SD for each evaluated condition (n = 8). *p ≤ 0.05, Student T-test. One representative experiment of 4 independent ones performed.

*Salmonella* quantification after MET release revealed that approximately 10% of the initial inoculum died after 60 minutes of infection (p = 0.0034, Student T-test) (**Figure 6**). No bacterial death was observed when DNAse I pretreatment was applied, suggesting that MET dismantling impairs bacterial death. Hence, the above mentioned GPF dimming could be attributed to a decreased GFP active expression by dying bacteria, most likely due to the action of enzymes present within the MET. Altogether, these observations reinforce our hypothesis that MET could trap and kill *Salmonella*.

**Figure 7:**
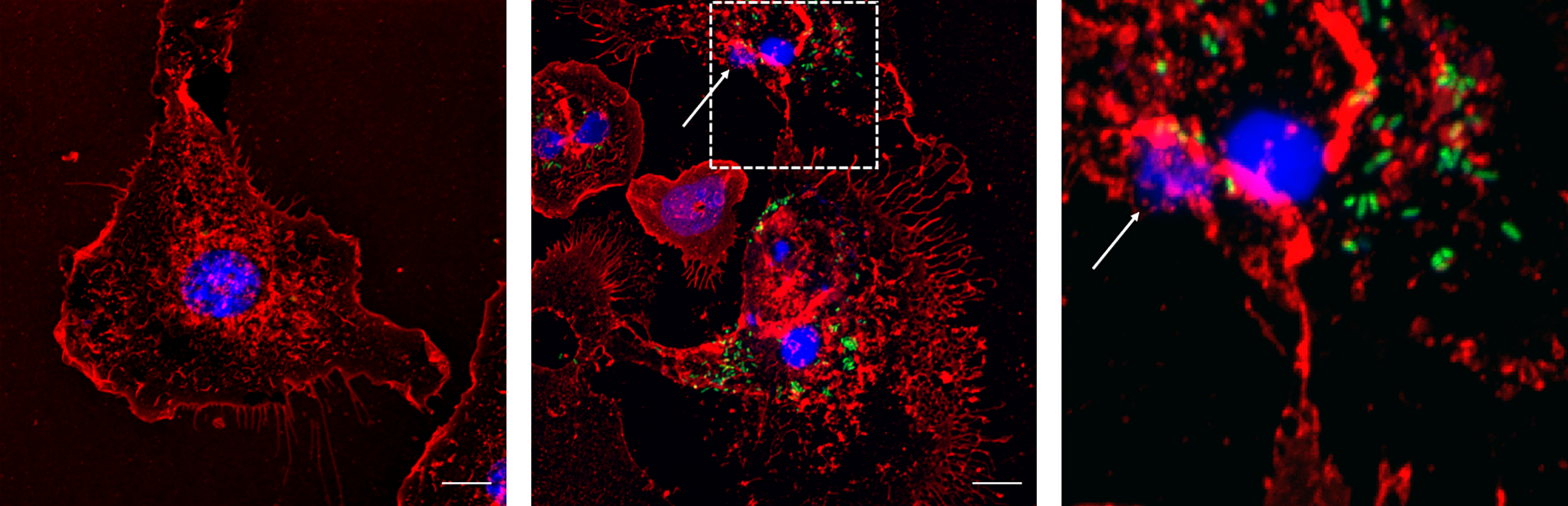
Bone marrow derived macrophages infected with *Salmonella enterica* serovar Typhimurium LVR01 (40:1). Left panel: Uninfected cells (Control). Center panel: 30 minutes of infection. Right panel: zoomed-in image from central panel (dashed square). The images are maximum intensity z-projections of all the slices in the stack in the three channels. In red: membrane, blue: DNA, green: *Salmonella*. The arrows point out the presence of METs. The scale bar represents 10 µm.

Finally, this phenomenon was confirmed in primary macrophages. MET release was also observed in bone marrow-derived macrophages infected with *Salmonella* as for J774A.1 macrophages (30 minutes, MOI 40:1) (**Figure 7**).

## Discussion

*Salmonella* is a major pathogen that still lacks efficient methods of control. Understanding the complexity of the immune response to *Salmonella* infection shall facilitate the development of improved vaccines and other tools to control the burden of *Salmonella* induced diseases. Here we demonstrated that MET release is part of this picture and contributes to killing of extracelullar bacteria. To date, MET release has been observed in response to many pathogens, including certain gram negative bacteria such as *E. coli*, *H. influenzae* and *K. pneumoniae*, amongst others. Hereby, we reported and characterized for the first time that *Salmonella* Typhimurium can induce METs. This event was observed as the extrusion of extracellular DNA fibers from macrophages, which are no longer visualized in the presence of DNAse I. The size of these fibers ranges from 10 to 50 μm approximately, which is in accordance with the reports of Brinkmann and Doster groups (Brinkmann et al., 2004; Doster et al., 2018).

The addition of live *Salmonella* to J774A.1 macrophage cultures induced METs release within 10 minutes. Our results are in line with those from Hellenbrand and Muñoz-Caro groups, which reported MET release triggered by other pathogens in less than 30 minutes (Hellenbrand et al., 2013; Munoz-Caro et al., 2014). The original description of NETs showed a rapid release of extracellular DNA (10 minutes after stimulation with PMA), but latter reports of the same group have suggested ETosis could take several hours (Brinkmann et al., 2004; Fuchs et al., 2007). In this case, one hour after infection, most macrophages were no longer viable, indicating that MET formation involves cell death. Nevertheless, it has been described that in certain circumstances vital ETosis could also occur (Byrd et al., 2013; Pilsczek et al., 2010; Rochael et al., 2015). The potential benefit of macrophage suicidal METosis could be related to *Salmonella* ability to survive inside them in SCV. In this way, two complementary functions against *Salmonella* could be taking place: to retain and kill extracellular bacteria within METs and also to avoid invasion and survival of bacteria inside macrophages, through self-killing. Indeed when infection is carried out at higher bacterial load (i.e. MOI 100:1), MET formation is also detected and macrophages are severely damaged (**Figure S2**). However, surviving macrophages contain bacteria trapped inside SCVs (**Movie SM2**).

Further analysis reveals that *Salmonella* trapped within these METs display restricted movement when compared to free swimming bacteria. In addition, once movement is restricted due to entrapment within MET bacteria die, as evidenced by GFP fluorescence dimming. The antimicrobial activity of ETs has been mostly attributed to enzymes such as elastase or myeloperoxidase; nevertheless, the antimicrobial effect of histones themselves has been reported decades ago (Hirsch, 1958), and DNA has been particularly linked to NET bactericidal activity more recently (Halverson et al., 2015). Since we did not define the MET composition besides DNA, we are not able to provide more information about the precise antimicrobial mechanism against these particular bacteria. Further studies are needed in order to define the nature of this new anti-*Salmonella* phenomenon. Zhao et al. (2018) have reported that BCA-enhancement of MET release is an efficient way to eliminate extracellular *Salmonella*, either *in vitro* or *in vivo*, therefore reinforcing our results that pose this as a bactericidal mechanism. We noticed a discrete bacterial killing by MET that could not be explained by phagocytosis, suggesting that *Salmonella*-induced METs have effective but limited antimicrobial capacity. This is in accordance with a previous report for *E. coli*, a model enterical pathogen, which proposed a role for METs on limiting dissemination rather than directly killing extracellular bacteria (Liu et al., 2014). The potential physiological role of ET release as an important strategy against pathogens has been recently reviewed (Ravindran et al., 2019). Here we show the following sequence of events: 1) *Salmonella* triggers rapid MET release, 2) bacteria are trapped within these METs and die, and 3) macrophages die.

Different stimuli have been reported to trigger MET release [reviewed in (Doster et al., 2018)] while the precise mechanisms are poorly understood. The particular signals that elicit METs formation upon *Salmonella* infection are still unknown. It has been described that amyloid fibers trigger NET release (Azevedo et al., 2012). *Salmonella*, as other enterobacteria, produces curli fibers, which are bacterial amyloids. These fibers are potent inductors of an inflammatory response and are involved in early stages of cellular aggregates and biofilm formation (Barnhart and Chapman, 2006). Since LVR01 is a biofilm producing strain, it is then possible that *Salmonella* fimbria curli could be inducing MET release.

To sum up, we observed for the first time that *Salmonella* Typhimurium LVR01 triggers MET release under experimental conditions, which subsequently leads to bacterial clearance and macrophage death. Further studies are needed to fully address the relevance of this mechanism in immunity against *Salmonella* as well as the precise molecular mechanisms involved.

## Data Availability Statement

The raw data supporting the conclusions of this article will be made available by the authors, without undue reservation.

## Ethics Statement

The animal study was reviewed and approved by Comisión Honoraria de Experimentación Animal (CHEA), Facultad de Medicina, Uruguay.

## Author Contributions

AM, MM, and PS contributed to the conception and design of the study. AM and MM performed the experiments. PS, NC, JJ-W, and SH obtained, processed and analyzed confocal images. JAC contributed to interpretation of the data. AM wrote the first draft of the manuscript. All authors contributed to the article and approved the submitted version.

## Funding

This work was supported by the Chilean Millennium Science Initiative P09-015-F; FONDECYT 1181823; FONDECYT 1211988; DAAD 519605, CORFO 16CTTS-66390 and ACM 170003 to SH, PS, JJ-W, NC-H; AUCI Program for South-South Collaboration Uruguay-Chile.

## Conflict of Interest

The authors declare that the research was conducted in the absence of any commercial or financial relationships that could be construed as a potential conflict of interest.

## Supplementary Material

Supplementary Figure 1 Sterile MET release evaluation. Confocal microscopy images of J774A.1 macrophage extracellular trap release at t = 60 minutes post incubation with supernatant of other culture. Left: Control, J774A.1 cells cultured with fresh medium. Center: J774A.1 cells post incubation with cell supernatant. Right: J774A.1 cells cultured with cell supernatant, pre-treated with DNAse I. WGA staining is shown in red and DNA in blue. The arrows point out MET release. The scale bar represents 10 µm.

Supplementary Figure 2 MET release after *Salmonella* infection at higher bacteria load. *Salmonella* enterica serovar Typhimurium LVR01 infection of J774A.1 macrophages at t = 10, 30, and 60 minutes post exposition to bacteria at a MOI 100:1. Maximum intensity z-projections are shown for membrane visualization with WGA staining (red), DNA (blue), and *Salmonella* (green). The arrows point out MET release. The scale bar represents 10 µm.

Movie 1
**(Still image 1)**. *Salmonella* retention within MET and subsequent bacterial death. Confocal microscopy with time-lapse imaging of J774A.1 macrophages infected with *Salmonella* enterica serovar Typhimurium LVR01 30 minutes post infection (63X magnification, 1 frame captured every 0.57 seconds, video rendered at 25 frames/sec). In red: membrane, blue: DNA, green: *Salmonella*. The scale bar represents 5 µm.

Movie 2
**(Still image 2)**. Tracking analysis of *Salmonella*. Each bacteria was tracked and represented by a colored dot, with its corresponding trajectory traced by a line of the same color. Bacteria that were trapped within the MET had a constrained movement inside the MET area.

Movie SM1
**(Still image movie S1)**. *Salmonella* within a MET with a different look-up table (LUT). Movie 1 was processed so the emission color of channel was changed to a red/green LUT. High fluorescence intensity values are observed in green and low intensity values in red. The same LUT was applied for both green and blue channels (for GFP and DNA, respectively).

Movie SM2
**(Still image movie S2)**. *Salmonella* inside *Salmonella* containing vacuoles (SCV). Confocal microscopy with time-lapse imaging of J774A.1 macrophages infected with *Salmonella* enterica serovar Typhimurium LVR01, MOI 100:1, 30 min post infection (63X magnification, 1 frame captured every 0.49 seconds, video rendered at 5 frames per second). Membrane WGA staining (red), DNA (blue), and *Salmonella* (green). The scale bar represents 5 µm.

## Abbreviations

NET, Neutrophil Extracellular Traps; MET, Macrophage Extracellular Trap; SCV, *Salmonella* containing vacuole; PAMP, Pathogen-Associated Molecular Pattern; ET, Extracellular Trap; GFP, Green Fluorescent Protein; DMEM, Dulbecco’s Modified Eagle’s Medium; FBS, Fetal Bovine Serum; BMDM, Bone Marrow Derived Macrophages; RPMI, Roswell Park Memorial Institute; M-CSF, Macrophage Colony-Stimulating Factor; MOI, Multiplicity of Infection; PMA, Phorbol Myristate Acetate; LBA, Luria Bertani Agar; PBS, Phosphate-Buffered Saline; WGA, Wheat Germ Agglutinin; GSDM-D, Gasdermin D; OMV, Outer Membrane Vesicle.
