# Supplementary figures and images for: Salmonella Typhimurium Triggers Extracellular Traps Release in Murine Macrophages
Source: Front Cell Infect Microbiol. 2021 Apr 26;11:639768. doi: 10.3389/fcimb.2021.639768 (PMC8107695; doi:10.3389/fcimb.2021.639768)

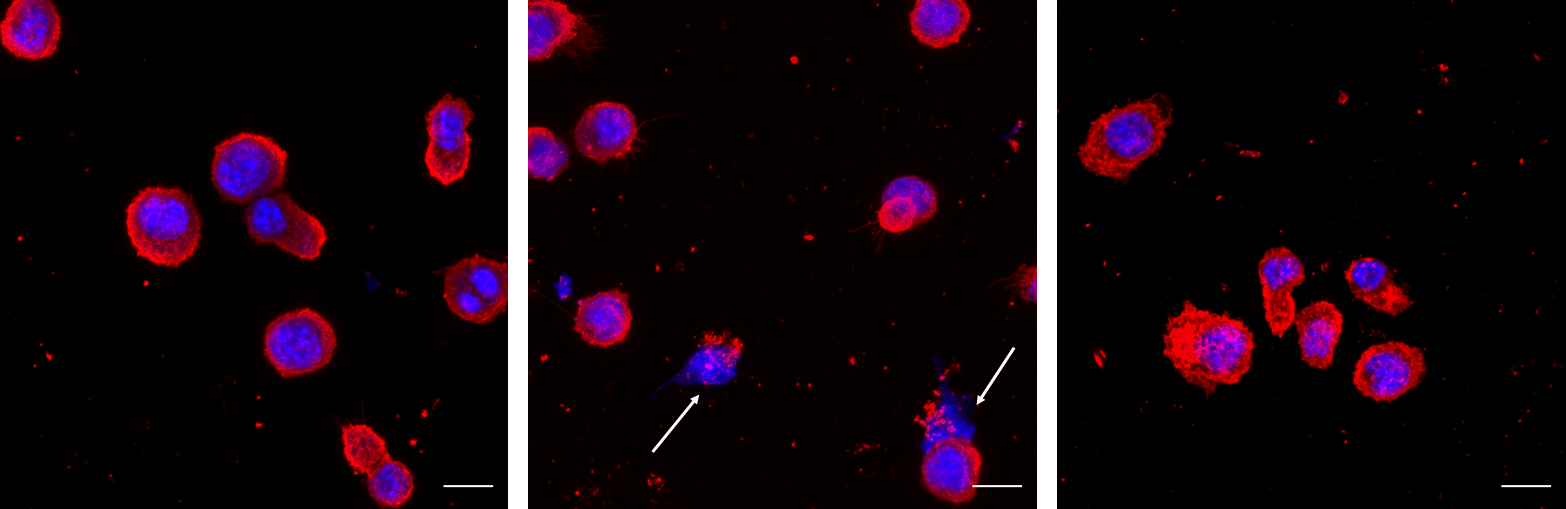

Supplement: Supplementary Figure 1 — Sterile MET release evaluation. Confocal microscopy images of J774A.1 macrophage extracellular trap release at t = 60 minutes post incubation with supernatant of other culture. Left: Control, J774A.1 cells cultured with fresh medium. Center: J774A.1 cells post incubation with cell supernatant. Right: J774A.1 cells cultured with cell supernatant, pre-treated with DNAse I. WGA staining is shown in red and DNA in blue. The arrows point out MET release. The scale bar represents 10 µm. [file Image_1.tif]

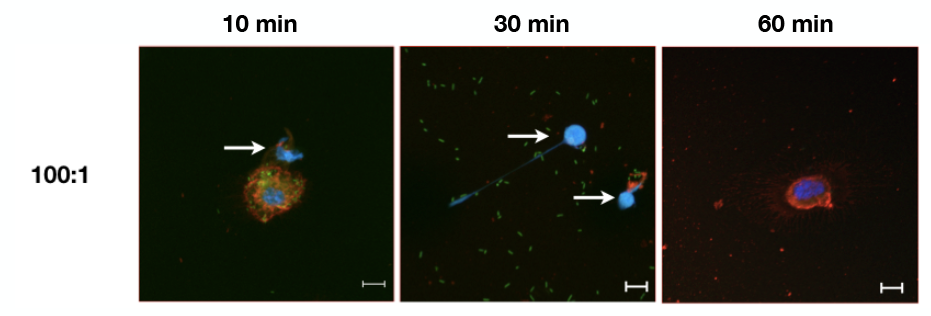

Supplement: Supplementary Figure 2 — MET release after Salmonella infection at higher bacteria load. Salmonella enterica serovar Typhimurium LVR01 infection of J774A.1 macrophages at t = 10, 30, and 60 minutes post exposition to bacteria at a MOI 100:1. Maximum intensity z-projections are shown for membrane visualization with WGA staining (red), DNA (blue), and Salmonella (green). The arrows point out MET release. The scale bar represents 10 µm. [file Image_2.tif]
